# Willingness to participate in a personalized health cohort – insights from the swiss health study pilot phase

**DOI:** 10.1186/s12889-024-19650-z

**Published:** 2024-08-07

**Authors:** Nolwenn Bühler, Annika Frahsa, Nathalia González Jaramillo, Réjane Morand Bourqui, Semira Gonseth Nusslé, Claire Zuppinger, Murielle Bochud, Natalie von Goetz

**Affiliations:** 1 Institute of Social Sciences, University of Lausanne, Lausanne, Switzerland; 2 Institute of Social and Preventive Medicine, University of Bern, Bern, Switzerland; 3 Federal Office of Public Health, Schwarzenburgstr. 157, Bern, 3003 Switzerland; 4 Center for Primary Care and Public Health, Unisanté, University of Lausanne, Lausanne, Switzerland

**Keywords:** Scenario-based approach, Informed consent, Attitudes towards research, Trust in science, Biomonitoring

## Abstract

**Background:**

This paper explores the feasibility of establishing a large-scale population-based cohort and biobank in Switzerland by assessing potential participants’ needs, expectations, and concerns about such an infrastructure providing information on health, lifestyle, and exposure trajectories, the development of disease, and risk factors over time.

**Methods:**

We utilized a scenario-based questionnaire in the Swiss Health Study pilot phase (2020–2021), involving 1349 adults aged 20–69 from the cantons Vaud and Bern. We conducted descriptive statistics supported by R and qualitative content analysis of *n* = 374 open responses related to attitudes towards research.

**Results:**

We highlight the benefits and challenges of the scenario-based approach, discuss the sample represented in the pilot phase, and present implications for building a full cohort. We also report on participants’ attitudes towards and previous experience with health research. We analyze references regarding informed consent and feedback, attitudes towards the Swiss Health Study, and recommendations on improving its scope, design, and instruments. Results indicate a high interest (90%) in participating in a national health study, with 85% of a random population sample willing to join a long-term cohort. Only 43% were familiar with biobanks, and 44% preferred general consent. Trust was high for Swiss-based public research but lower for researchers from other countries or private sector. Over 95% expressed willingness to complete online questionnaires, undergo physical examination, and donate biosamples. Almost all participants wanted to know the outcomes of the medical tests (99.5%) and the exposure to environmental stressors (95%) from their study center visit. Preferred tools for monitoring sleep, physical activity, and diet were known smartphone apps with automatic data management.

**Conclusion:**

Overall, the study reveals a positive attitude towards personalized health research, with a strong willingness to share data and samples. Key insights focus the meaning of informed consent for participation, the relevance of sampling and representativeness, as well as the significance and challenges of personalized feedback, especially regarding environmental health concerns. Findings emphasize participants’ supportive yet reflexive stances, underscoring the importance of aligning research values with individual values in personalized health research. These insights contribute valuable considerations for refining the scope, design, and instruments of future cohort studies.

**Supplementary Information:**

The online version contains supplementary material available at 10.1186/s12889-024-19650-z.

## Background

Personalized or precision medicine initiatives seek to enhance medical interventions by leveraging various data types, including biological information and biomarkers [1]. These initiatives aim at understanding causal mechanisms and interdependencies [2] and tailoring health promotion and prevention interventions as well as early disease detection [3, 4] relying on extensive datasets and biobanks.

In Switzerland, entities like the Swiss Personalized Health Network (SPHN), the Swiss Biobanking Platform (SBP), Health 2030, or the Swiss Institute of Bioinformatics (SIB) promote data-driven approaches, aligning with (inter-)national regulations for data privacy and sharing [5, 6]. So far, a large part of this infrastructure is directed toward facilitating the secondary use of routinely collected hospital-based data for research (SPHN). Ideally, such an infrastructure would also facilitate the efficient and safe use of data providing information on health, lifestyle, and exposure trajectories, the development of disease and risk factors over time, if conducted in parallel to a large-scale population-based cohort and biobank. It would also allow identifying determinants of healthy aging, causes of disease or contributing factors, health services access, use and costs, which are crucial to developing public health strategies and policies for the improvement of population health [7].

However, Switzerland lacks a centralized health database [8] and a large-scale national biobank [9], such as e.g. the UK biobank [10], as well as the legal basis for data linkages like in Finland [11–13]. Existing initiatives like the Swiss National Cohort (SNC) [14, 15] lack biological samples and comprehensive data linkage. A few population-based cohorts have been set up during the past 30 years and are considered to be very successful, such as SAPALDIA [16, 17], CoLaus-PsyCoLaus [18–20] or the Specchio-Covid-19 cohort study [21]. However, these local initiatives do not comprehensively cover Switzerland and have a limited sample size (< 10’000 participants).

Setting up a nationwide cohort and biobank in Switzerland is strategically important [7]. Yet, participation rates in such initiatives have declined. Public preferences on data management and governance highlight the need for personalized approaches and transparent consent processes [22, 23].

Within the specific Swiss cultural and political context, understanding the interactions of previous knowledge on health research and trust on the one side, and the willingness to participate and provide data and biological samples on the other, has become a key concern. In alignment with international ethical standards aiming to protect the rights of people participating in research, so-called public participation surveys and initiatives have flourished in this domain and become a standard for the good governance of health research [24]. These initiatives assume that consulting or involving the “public” can help design and implement research processes that not only take their preferences and expectations into account but also attain wide acceptance from society, funders, and donors.

Studies have indicated a positive attitude towards personalized health research among Swiss residents [22], driven by altruism and a sense of contributing to the overall greater good [25, 26]. At the same time, concerns persist over data privacy, [23] commercial use of data [26], and limited enrolment opportunities [25].

To address these concerns and assess willingness for participation, we aimed to develop a scenario-based survey to create a narrative around the survey questions, considering both respondents’ potential lack of knowledge or opinions on these perspectives [27], as well as to make notions and implications of research participation less abstract and closer to daily life [28, 29].

This paper will, thus, (i) present a new instrument to assess citizens’ willingness to participate in personalized public health cohort research, (ii) identify critical issues regarding participation in such a cohort, and (iii) present initial results from the Swiss Health Study – pilot phase (SHeS - pilot)on participants’ attitudes, knowledge of informed consent, and motivations for engagement, offering insights into methodological refinement for future studies.

## Methods

### Setting

In response to parliamentary motions asking the government to act on the impact of pollutants on health, the Federal Office of Public Health (BAG) initiated an evaluation of existing cohorts with human biomonitoring (HBM). Based on this evaluation and various exchanges with the research community, the Swiss Federal Council endorsed the SHeS-pilot [8]. The purpose was to lay the basis and test the feasibility of a national population cohort with HBM. The objectives of this Swiss Health Study (SHeS), if continued and as conceived in the pilot, are the following: (a) a *surveillance goal*, to conduct human biomonitoring and investigate sources of exposure to environmental contaminants, dietary intakes, and nutritional status of the population as well as other types of exposure; (b) a *scientific goal*, to advance research on the exposome, gain a better understanding of health determinants and the burden of diseases, and identify relevant exposure biomarkers; and (c) a *public health policy goal*, to support informed decision-making when developing health policies and to evaluate the impact of public health interventions, at both regional and national levels.

### Questionnaire development

Drawing on the combined expertise of a molecular epidemiologist and a socio-anthropologist working on the SHeS-pilot, we developed a scenario-based questionnaire for assessing the attitude of participants towards health research, common practices, and preferences (cf. the final questionnaire with all questions Supplementary online S1). Through the plotting of the questions and immersion in a concrete situation, respondents were invited to put themselves in these situations when answering the scenario-based questionnaire. Our goal was to bring respondents closer to what it would entail to participate in a cohort and, to some extent, bring back the social context erased by more abstract questions. We discussed at length several “contexts” to unfold a realistic scenario. We opted for one deemed implementable in a national cohort. The setting consists of a clinical encounter between a general practitioner, who could also be another healthcare professional, and a citizen/patient.

**Fig. 1:**
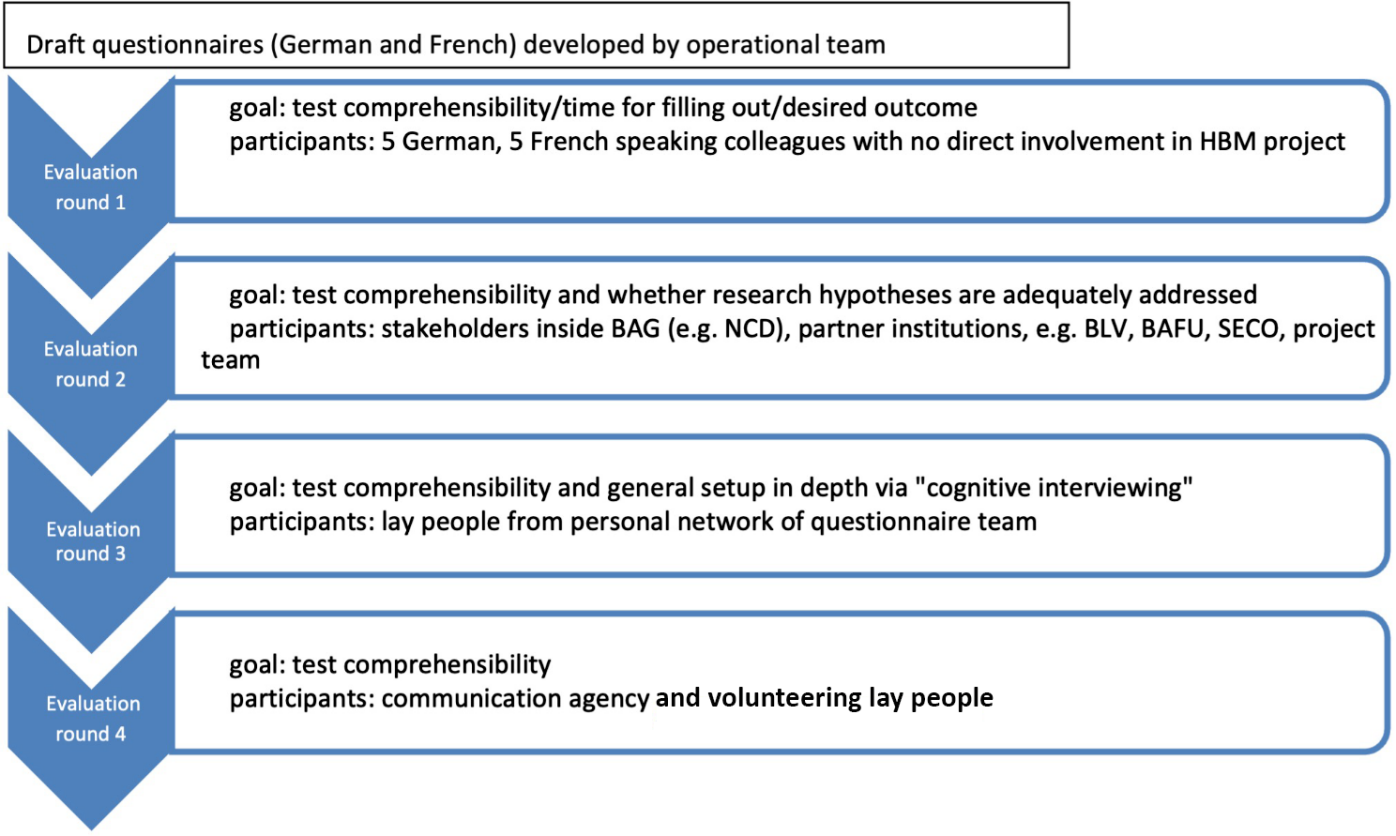
Overview on questionnaire development

Apart from the scenario-based questionnaire, we used standardized survey instruments to assess health status, lifestyle and behaviors, and exposure determinants such as housing. To pre-test and adjust questionnaires, the team performed four evaluation loops (cf. Figure 1). During evaluation loops, team members were present to document feedback in an unstructured way for each item and each participant. The team discussed changes proposed and adjustments needed and agreed upon within the project team after each loop. In evaluation round 1, ten persons from a convenience sample of scientists, comprising co-authors’ colleagues not directly involved in the project, checked the German and French questionnaires for understandability, user-friendliness (e.g., formatting), correct translation, and time for filling in the questionnaires. In evaluation round 2, scientific staff from partner agencies and academia evaluated suitability of the questionnaires against the underlying research hypothesis. The team created and shared a database for comparison and checking with evaluators for each of the questions to its respective research hypothesis, expected answers in both languages, and the origin of questions (if adapted from other questionnaires). In evaluation round 3, cognitive interviews were conducted with seven laypeople to ensure that survey participants would understand the questions in the intended way. For this, they answered the questions in the presence of the interviewer and explained how they understood each question in their own words. In case this was not in the intended way, the interviewer noted the discrepancy. In evaluation round 4, a communication agency and five volunteering citizens (self-assigned from the general public) checked adjusted questionnaires to ensure comprehensibility for lay people of the final version.

The final scenario-based questionnaire included 11 questions about socio-demographics and 31 questions about knowledge of and attitudes towards health research (S1). The scenario-based approach aimed to allow respondents to project themselves in situations they could easily relate to and progressively uncover a specific understanding of a concept rathern than providing definitions at the beginning of the survey.

The questionnaire also featured open comments fields, providing room for qualitative responses to the survey on willingness to participate, which can help further develop the questionnaire for a follow-up study. In the present manuscript, we analyzed the results from the pilot phase related to the knowledge of and attitudes toward health research.

### Sampling

The SHeS-pilot (a cross-sectional study on adults aged 20–69 between January 2020 and December 2021) used a mixed sampling method [30] reported in detail elsewhere [8] that included [1] *a random sample* of participants from the general population, aged 20 to 69 years, of the cantons of Vaud and Bern, [2] a *purposive sample* of participants following a vegan or vegetarian diet for at least one year for a selenium sub-study, and [3] *a self-selected sample*, with Swiss residents aged 20 to 69 years eligible to fill in the study questionnaires spontaneously on the study website.

To account for possible differences in the results due to the sampling method or level of participation, we present the descriptive results of our study for a total sample (*n* = 1349), distinguishing participants who completed study questionnaires only (*n* = 560), i.e., *partial participation* in the following, and for study participants who completed both questionnaires and a visit to the study centre from the random and purposive sample (*n* = 780), i.e. *full participation* in the following. Study centres were located in the Lausanne University Hospital and the Berne University Hospital (Inselspital). Visits took about 2.5 hrs and covered diverse assessments (medical history, lifestyle, cardiovascular, anthropometrics, body composition, frailty, respiratory tests) and the provision of various biospecimens (blood, urine).

Full details on sampling, study visits, and initial results are presented elsewhere [8].

### Data analysis

#### Qualitative analysis of open responses

We conducted a deductive qualitative content analysis, following Mayring [31], of the *n* = 374 responses to the open-ended comment box in the questionnaire. We used Microsoft Excel to support data coding. Three coders (AF, NB, and a student assistant, both trained and experienced in qualitative data analysis) carried out open coding of all responses. For the trustworthiness of findings, we met to discuss and agree upon discrepancies and ambiguities in how we conceived and applied codes. We organized codes into categories derived from topics covered in the questionnaire, such as willingness to participate in a personalized health cohort, knowledge and perception of cohort health research, and other issues raised, such as comments on the concrete execution of the SHeS-pilot. Each response could have multiple codes. During the analysis process, we also held repeated meetings with co-authors to discuss codes and categories and to agree on the final set of codes and the category system (cf. for a thematic map of the category system Supplementary file S2). We created two main categories: [1] participation in health research in general and [2] participation in the SHeS-pilot with nine sub-categories (cf. in detail S2):

• Perceived challenges in personalized health research.

• Perceived opportunities in personalized health research.

• Perceived limitations of personalized health research.

• Participation in SHeS (readiness, concerns, reasons for non-participation).

• Expectations from SHeS (return of results, evidence on effectiveness of preventive medicine approaches).

• Participants’ general opinion about SHeS.

• Set-up of SHeS (requests for opt-out options, monitoring devices and software, requests for clarity on procedures).

• Set-up of questionnaires (Improvements in content, form, wording, general comments).

• Feedback on scenario approach taken.

#### Statistical analysis

We calculated descriptive statistics for all participants; normally distributed variables were described by the mean and standard deviation (SD). Non-normally distributed variables were described by the medians and interquartile ranges (IQR). All categorical variables were described by absolute and relative frequencies. Chi-squared analyses were used to compare the demographic characteristics according to the sampling method. We determined the internal consistency of the questionnaire by comparing the questions against one another, using Cronbach’s alpha [32]. For the analysis, we excluded *n* = 9 participants from the self-selected sample who also completed a study visit from our comparative analysis regarding study participation levels.

We present socio-demographic characteristics according to the different sampling methods.

## Results

In the results section, we will (i) analyse the scenario approach taken in the questionnaire to assess willingness to participate in a national cohort, (ii) critically reflect on the sample represented in the pilot phase and implications for willingness to participate, present (iii) participants’ willingness to participate, their attitudes towards and previous experience with cohort-based health research, (iv) their preferences regarding informed consent and feedback, as well as (v) their attitudes towards a potential national cohort health study and recommendations on how to improve the scope, design, and instruments of its pilot for a successful implementation at a larger scale.

### The scenario approach taken

We built our scenario on an appointment with a general practitioner, easily extendable to any healthcare professional. While there currently is a tendency not to have a general practitioner (GP) in Switzerland [33], it remains a privileged contact zone with the health system. GPs, and more generally, primary healthcare professionals, are usually not involved in scientific research. Still, for a national cohort, they might be relevant partners, e.g., in the recruitment of participants [34]. The proximity with a primary healthcare professional also helped to make the scenario realistic, personalizing the contact and creating a space for addressing the individual and more general implications of research participation. This helped to construct a narrative where information and questions are progressively released, allowing people to voice their opinions after being sensitized to some of their implications.

Some participants suggested adaptations to the developed scenario-based approach.

While respondents generally commented positively about the scenario approach, some stated they preferred questions linked to their perspective/person/name instead of imagining to be someone else.

I would leave out the part about the fictitious Mrs. Müller or formulate it in such a way that the answers are given from a personal point of view. All questions should refer to the person of the study participant.

### Critical reflection of the sample composition in the pilot phase

The socio-demographic characteristics of the participants from the different samples are depicted in Table 1. They are compared with cantonalresident data from the Federal Statistical Office for.

**Table 1:**
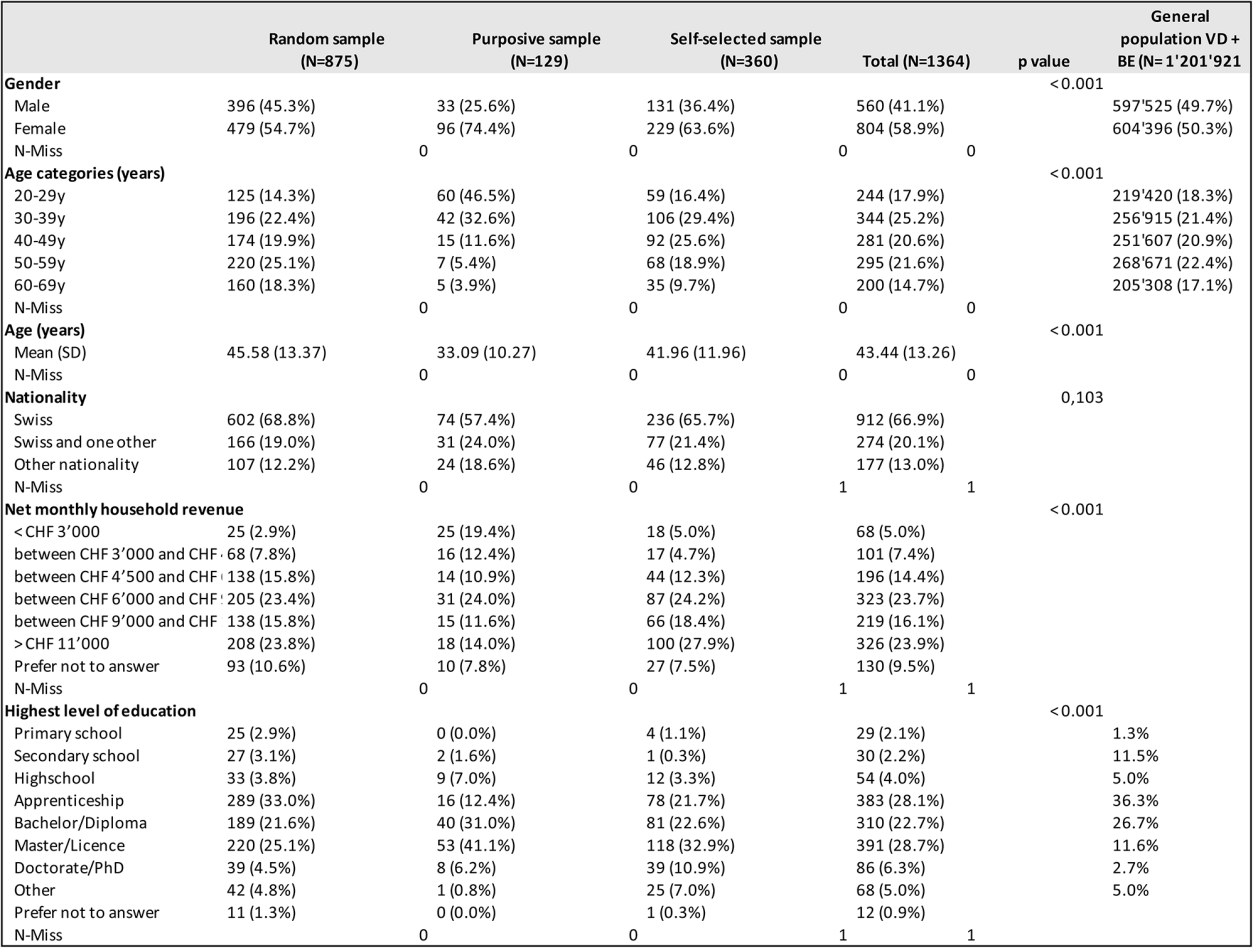
Socio-demographic characteristics of participants according to sample type

Participants’ characteristics from the different samples differed in various characteristics. Women were over-represented in all samples. The mean age and the age distribution of the participants in the random sample were similar to the ones of the general population aged 20 to 69 years. Participants from the purposive sample, following a vegetarian or vegan diet, were, on average, ten to twelve years younger than participants from the random and self-selected sample and the general population in the Swiss cantons Vaud and Bern. A statistically significant difference existed concerning education levels in all SHeS-pilot samples and the distribution of educational levels among the general population in the two cantons.

Regarding the total of 374 open response fields and comments in the questionnaire, the purposive sample commented more often than the other groups. 30% (*n* = 39) from the selenium sub-study group commented on the questionnaire, whereas in the random and the self-selected samples 20% (*n* = 175) and 10% (*N* = 36) commented at least once, respectively.

### Willingness to participate and attitudes towards health research

The interest in participating in a national study on health was high to very high for most participants (cf. Table 2). The willingness to participate (yes/maybe) in a long-term cohort study was also high to very high among a vast majority: 87.0% (*n* = 682) among the complete participation group, 91% (*n* = 512) among the group who filled out online questionnaires only (cf. Table 2).

There were few statistically significant differences concerning associations in motivations to participate and different socio-demographic variables (cf. details Supplementary file S3). Getting a free medical check-up was strongly age-association, with younger participants being much more motivated by this incentive than older participants (80.7% among the 20–29 years to 55.0% among the 60–69 years, P for trend < 0.001 in the random sample with similar results for the two other samples). The same was found for financial rewards and small gifts as motivations by young people than by older ones (P for trend < 0.001 in all samples) [8]. In the random sample, participants with higher educational levels (Master, Licence/Doctorate/PhD) were more motivated by their wish to contribute to the progress of medicine and their interest in research and health than people with lower educational levels (85.7–71.5% among people with apprenticeship and 71.8% among people with primary education). We did not find statistically significant differences according to gender or nationality.

In open comments, though, several participants expressed concern about the long-term commitment a cohort study would require.

*Interest in participating*,* but limited by reality (work*,* children…) So commitment to be assessed on a case-by-case basis according to my availability at the time.*

They would be willing to participate for a given period, such as several months, but are reluctant to commit themselves longer, often due to multiple other commitments at home or work, as indicated by the open comment above.

**Table 2:** Attitudes, knowledge, opinions, and willingness to participate in personalized health research

Variables	Total sample *N* = 1340 No. participants (%)	Complete participation group *N* = 780 No. participants (%)	Partial participation group *N* = 560 No. participants (%)	*P* value for comparison
Attitudes towards health research				
Previous participation in a health study	128 (10)	63 (8)	65 (12)	0.000*
***Previous knowledge about ethics committees***				
Know and understand what EC are	659 (35)	382 (36)	313 (37)	0.003*
Know that EC exist but do not understand how they work	429 (38)	267 (28)	162 (39)	
No previous knowledge	215 (16)	131 (17)	84 (15)	
***Previous knowledge about biobanks***				
Know and understand what biobanks are	577 (40)	311 (33)	266 (41)	0.05
Know that biobanks exist but do not understand what they are	449 (28)	278 (27)	171 (42)	
No previous knowledge	313 (23)	191 (24)	122 (22)	
***Opinion towards health studies (First impression)***				
Very much in favor	595 (43)	315 (40.4)	281 (44)	0.09
In favor	668 (44)	417 (53.5)	251 (45)	
Against	39 (3)	26 (3.3)	12 (2)	
Very much against	21 (2)	1 (0.1)	0 (0)	
Not interested in health studies	15 (1)	21 (3)	15 (3)	
***Willingness to participate in a long-term health study***				
Yes	537 (33)	273 (29)	264 (46)	0.009*
Maybe	657 (36)	409 (35)	248 (45)	
Maybe not	126 (10)	85 (11)	41 (7)	
No	18 (1)	13 (2)	5 (1)	
***Opinion towards genetic studies (First impression)***				
Very much in favor	450 (28)	260 (47)	190 (28)	0.000*
In favor	719 (48)	420 (48)	299 (48)	
Against	113 (8)	65 (8)	48 (9)	
Very much against	11 (1)	6 (1)	5 (1)	
Not interested in genetic studies	45 (4)	28 (4)	17 (3)	
***Willingness to participate in genetic studies***				
Yes	1004 (75)	578 (74)	426 (76)	0.91
No	46 (3)	24 (3)	22 (4)	
Not sure	288 (22)	177 (23)	111 (20)	
***Willingness to participate according to required actions***				
Answering a detailed questionnaire about risk factors	1320 (99)	773 (99)	547 (98)	0.09
Physical examination at a medical center	1286 (96)	758 (97)	528 (94)	
Giving blood, urine, or saliva samples	1280 (96)	753 (96)	527 (94)	
***Opinion towards the creation of a national Swiss biobank***				
Very much in favor	536 (33)	299 (31)	237 (49)	0.51
In favor	595 (45)	358 (50)	237 (49)	
Against	50 (4)	32 (4)	18 (3)	
Very much against	12 (1)	6 (1)	6 (1)	
Not sure	5 (11)	85 (11)	61 (11)	
***Opinion towards the importance of studying the effects of the environment on health***				
Very important	933 (70)	517 (66)	416 (75)	0.21
Important	379 (51)	250 (38)	129 (23)	
Not very much important	16 (1)	8 (1)	8 (1.4)	
Not important at all	1 (0.1)	1 (0.1)	0 (0)	
Not interested in such a study	8 (0.6)	3 (0.4)	5 (1)	
***Type of preferred informed consent***				
General consent	578 (40)	344 (45)	234 (49)	0.81
Specific consent	406 (52)	236 (52)	170 (52)	
Dynamic consent	285 (21)	163 (21)	122 (22)	
Not sure	46 (3.4)	26 (3.3)	20 (4)	
***Type of preferred biological samples to donate***				
Saliva	1275 (95)	750 (96)	525 (94)	0.06
Hair	1235 (92)	720 (92)	515 (92)	
Urine	1276 (95)	747 (96)	529 (95)	
Blood	1272 (95)	744 (95)	528 (94)	
Stools	894 (67)	510 (65)	384 (69)	
DNA	1016 (76)	588 (75)	428 (76)	
None	25 (2)	11 (1.4)	14 (2.5)	
***Type of preferred researchers to allow access to data***				
Researchers from Swiss institutions	1302 (97)	761 (98)	541 (97)	0.19
Researchers from foreign institutions	604 (43)	346 (45)	258 (50)	
Researchers from federal offices	973 (73)	553 (71)	420 (75)	
Researchers from non-profit organizations	998 (75)	577 (74)	421 (75)	
Pharmaceutical industry	352 (26)	199 (26)	153 (53)	
Agro alimentary industry	337 (25)	199 (26)	138 (25)	
Fitness industry	253 (19)	138 (18)	115 (21)	
None	19 (1.4)	11 (1.4)	8 (1.4)	

#### Attitudes towards health research and biomonitoring

Participants from the different samples and with varying levels of participation were comparable in most attitudes toward research. Overall, 95% (*n* = 1263) of the participants were in favour, or very much in favour, of health research in general, 89% (*n* = 1169) were very much in or in favour of genetic studies, 84% (*n* = 1131) were in favour of creating a national Swiss biobank, and 98% (*n* = 1312) considered that it is important or very important to study the effects of the environment on health (Table 2).

In the open comments, though, a few participants expressed concerns about the independence of science, particularly in capitalist societies. Others worried about the purpose and benefit of detailed biomonitoring, linking it to a lingeringambition to optimize health to its maximum. One open comment acknowledged human limits and aging and death as natural processes and pointed at the possibility that knowledge gain may not always be what participants want.

*I asked myself if we really want to find out everything. At some point we have to die*,* and that’s a good thing.*

Concerning previous experience and knowledge, 10% (*n* = 128) of all participants had previously participated in health research, 52% (*n* = 659) had prior knowledge about how ethics committees work, and 43% (*n* = 577) declared to know and understand what biobanks are.

### Preferences regarding consent, data sharing, and personalized feedback

#### Consent types and data sharing

44% (*n* = 578) of respondents preferred to sign a general consent, meaning that data and samples can also be used for further and future research. 30% (*n* = 406) of participants preferred to provide specific consent for the given study only. 21% (*n* = 285) preferred dynamic consent,, that would allow a consistent change in consent preferences and enrolment in future studies.

97% (*n* = 1302) of all respondents agreed to share data with researchers from Swiss institutions, 75% (*n* = 998) with researchers from non-profit organizations, 73% (*n* = 973) with researchers from federal offices, 45% (*n* = 604) with researchers from foreign institutions, 26% (*n* = 352) with the pharmaceutical industry, 25% (*n* = 337) with the food industry and 19% (*n* = 253) with the fitness industry. Only 1.4% (*n* = 19) would deny all access to their data (cf. Table 2).

Most respondents preferred their data and biological samples to be coded (85%, *n* = 1139) or anonymized (72%, *n* = 965). Nevertheless, one in every three participants would accept sharing non-coded data and non-coded biological samples.

Across all age groups, one of the main reasons for non-participation was fear of data misuse and concerns about data protection [8]. In the open comments, some participants expressed their reluctance to sdhare data and samples sharing outside of a specific study, mainly because of fear their data and samples being shared with private companies.

*I answered the questions about passing on samples/data to other researchers and about consulting my doctors rather cautiously*,* because for me it would depend very much on the question of the planned studies (e.g. passing on to researchers in the private sector) and data protection. E.g. a consultation of my physicians is probably not possible in anonymous form*,* i.e. my identity would be known to the researchers who consult my physicians. Therefore*,* I have chosen only ““possibly”” there.*

#### Attitudes towards receiving personalized feedback on findings

When participants answered about their preferences regarding feedback from the research data and results, a vast majority indicated that they would like to know the outcomes of the medical tests taken at the study centre (99.5%, *n* = 1333) and the outcomes regarding their environmental exposures (95%, *n* = 1273). Concerning genetic tests, the majority would like to receive feedback on results that may prove helpful to prevent or treat diseases (93%, *n* = 1246). However, 25% (*n* = 335) of the participants would refuse to know the results of genetic tests that could have possible repercussions on family members and eventual descendants.

### Attitudes towards and recommendations on how to improve the Swiss Health study

Participants provided various comments about the SheS-pilot and recommendations on how to improve it for the subsequent implementation of a national SHeS, covering issues such as study scope, design, instruments and tools used, or topics or even specific items they felt were missing.

#### Use of tools and instruments

Several comments referred to integrating widely known apps for health monitoring, such as sleep, physical activity, and food habits-tracking smartphone apps, into the cohort study rather than having to use specific instruments, such as an accelerometer, provided by the study center.

*There are already apps that can be used for this*,* e.g. NAME OF A BRAND*,* and also make automated data management possible. That would be digitalized research with more continuous data*.

*Why can’t existing measuring devices such as sports watches or smartwatches be included - data would already be available.*

Regarding integrating of health habit-tracking apps, participants pointed out that they would prefer apps with automatic data management rather than providing active input on those.

#### Topics under-represented or missing from participants’ perspectives

Participants also commented on topics and even concrete items that they felt were under-addressed or missing, among those (more) questions related to health behaviour, such as sport and physical activity, sleep, media use, or alcohol and substance consumption, prescription drugs but also exposure-related questions, such as noise or 4G/5G radiation.

Some participants called for integrating a positive definition and more comprehensive understanding of health into the study by asking questions about things that make someone happy.

*I’d find it interesting to add open-ended or multiple-choice questions*.

*(like “What makes you happy or unhappy?“).*

Others prefer an agency-centred perspective, e.g. by asking about illnesses or addictions people have overcome.

*I would find it good to be able to indicate not only what diseases you have ever had - but also whether/since when you have overcome them. For example*,* the eating disorder is quite typical for the teenage years and since then overcome - it’s a pity if something like that is then included in the statistics as a “permanent diagnosis”.*

### Improving the survey format, technical execution, and clarity of content

Almost 50 open responses referred to technical or format-related issues that participants experienced while completing the questionnaire. They provided detailed feedback on a various topics, including the the survey length, the questions’ clarity, programming mistakes, smartphone usability of the questionnaire, visualisation options, and the visibility/readability of items and survey elements. Participants also provided feedback on how to improve questionnaires in wording as well as reported on missing options for some questions.

*It’s also a pity that*,* when answering certain questions*,* you can’t see the choices (column headings) and you have to scroll back up to see them*,* then scroll back down to answer opposite the corresponding fields…*.

## Discussion

This study presented a new scenario-based questionnaire to assess willingness to participate in a personalised health cohort study. It built upon 1349 participants from the Swiss Health Study pilot phase to highlight the benefits and challenges of the scenario-based approach, discuss the sample represented in the pilot phase, present participants’ attitudes towards and previous experience with health research, and provide implications for building a full cohort. Results indicated a high interest (90%) in participating in a national health study, with 85% of a random population sample willing to join a long-term cohort. Only 43% were familiar with biobanks, and 44% preferred general consent. Trust was high for Swiss-based public research but lower for researchers outside the country or in the private sector. Over 95% expressed willingness to complete online questionnaires, undergo physical examination, and donate biosamples. 99.5% of participants wanted to know the outcomes of their medical tests, and 95% those of environmental exposure assessments at the study centre. Preferred tools for monitoring sleep, physical activity, and diet were smartphone apps, known and already in use, with automatic data management.

Open comments in the survey provided more insights into people’s perception of cohort health research in general, their willingness to participate, as well as recommendations on the concrete execution of the SHeS-pilot study. Only a few comments referred to the scenario-based set-up of the questionnaire. While the scenario-based approach generally was perceived as positive, some participants expressed they would have preferred to refer to themselves rather than an imaginary person and her journey through joining and participating in a cohort study. One aim of the scenario-based approach was to guide participants better through decisions about different consent forms. The idea was to be closer to real-life experience, processes, and potential impacts of a specific decision. However, open comments revealed that the issue of informed consent remained rather difficult to grasp and make decisions.

Our findings confirm previous studies in Switzerland regarding an overall positive attitude towards this kind of research and people’s willingness to share data and samples [22]. Like other studies, we found people to be concerned over data privacy and re-use and not to wish their data to be used by private companies [26]. The independence of science in this regard seems to represent an critical value.

Our study adds to the existing body of literature by providing critical insights into issues central for establishing a cohort in personalized health research in the Swiss context: [1] informed consent and types of consent, [2] participation or representativeness in a cohort study, and [3] the meaning and consequences of personalized feedback, especially regarding concerns about environmental health. It also sheds light on the critical and reflexive capacity of cohort participants, who while supporting the project, are also critical and share values that are important to them and should orient this kind of research.

### Participation in a cohort study: the issue of informed consent

While participatory approaches and the consultation of cohort participants during the development of questionnaires have become ‘good practice’ and part of the standards in health research governance [49], they often work as a *blank check* legitimizing ethically technoscientific endeavours without taking seriously or thoroughly exploring the knowledge, concerns and expectations of participants in those studies [40, 43, 45], neither addressing its politics [49]. Moreover, this kind of consultation procedure is often turned towards the scientists’ needs. It uses abstract and technical notions, which renders it challenging to assess to which extent respondents have understood the questions and, thus, how to interpret their answers. For example, the differences between general, dynamic, or regular informed consent are difficult to grasp for people unfamiliar with these ethical distinctions. In this study we aimed to frame both the consent and the described scenario-based questionnaire in everyday language, using examples and explanations for technical terms where necessary.

While general consent is often criticized for working as a blank check [36, 41], it is interesting to note that a large proportion of respondents is in favour of such type of consent, under the condition that they can withdraw it at any time. This might be interpreted as a sign of trust towards research institutions in Switzerland, although it remains unclear what trust means, how is it created and promoted, and what the nature of the greater good towards which people aim to contribute is [51, 53]. Indeed, qualitative research exploring practices of obtaining broad consent conducted within a Swiss university hospital showed the importance of institutional and relational factors in determining what can be described as “conditional” trust [39]. It is also important to unpack and deepen our understanding of what “altruism” and “greater good” actually mean for respondents/citizens living in a high-income country like Switzerland to shed light on the reciprocity logic at stake in personalized health research [38, 42, 52]. In the open responses, cohort participants commented on the ethics and politics of research, the values that should orient it, and what they expect in return for their participation and trust, such as the independence of science, the importance of the environmental determinants of health, and having agency over one’s own data.

At the same time, the high level of agreement with general consent might also indicate that the ‘ethics of research’ – in the sense of the moral values and ethical issues of research – as understood by cohort participants, are not so much at stake in the consent form, which has become a formal, very long and detailed document to comply with legal requirements and aiming at protecting institutions. In the open responses, participants pointed out the length and difficulty of the technical language of consent forms. One participant even said that there were so many precautions that it became suspicious. We explored this potential field of tension between trust and suspicion in more detail in a qualitative focus group study with SHeS-pilot participants elsewhere [38].

### Sampling in a cohort study or: the issue of representativeness

Compared to the general socio-demographic composition of Switzerland, the SHeS-pilot study showed a tendency towards higher participation of better educated and high-income citizens, an issue typical for health cohorts [44, 54] that leads to lower diversity regarding socio-economic status, migration experience or ethnic origin. Also, long-lasting cohort studies face the challenge of attrition bias in that the characteristics of those leaving the study differ from those remaining in the study [35].

What emerges from the participation patterns and participants’ comments is that the personal and financial “costs” of participating need to be addressed upstream. If the goal is to be very inclusive, the question of the time taken off work for research needs to be addressed, as well as specific means of recruitment. Moreover, the boundary between clinic and research, increasingly blurred in personalized health initiatives, remains important for most participants who make a clear distinction and do not want their data to circulate between these spheres.

### Receiving health outcome reports or: the issue of therapeutic misconception

Most participants expressed a high interest in receiving personalized feedback of study findings. While this might be perceived as a promising finding and a potential way to encourage cohort adherence, it also must be put into context. It probably reflects first the trend of personalized health relating to self-tracking and the growing social importance of health quantification and datafication [48, 55]. Secondly, the SHeS aims to conduct biomonitoring and, over the longer term, to identify environmental determinants of health. In Switzerland, the impact of chemicals and pollution on the environment is increasingly considered as a public problem and there are concerns in the population as the parliamentary motions at the base of this study indicate. In biomonitoring, the lines between so-called “environmental” and “lifestyle” risks might be blurred, as Washburn [56] has worked out. Therapeutic misconception has been coined to point to the challenging circulation of scientific and medical results between research and clinical care [57]. Empirical investigation of biobank research shows how ambivalent and blurry this boundary is in practice [58]. Participants’ great expectation towards scientific results, taken as medical, possibly actionable, information about oneself, relate to this phenomenon, and indicate how ambivalent their positions towards this boundary are: to be maintained for the sake of privacy and data protection, but to be blurred when it comes to the return of results.

### Strengths, limitations, and future research

Our study is, to the best of our knowledge, one of the first studies that developed a scenario-based questionnaire to assess people’s willingness to participate in personalized health cohort research, their knowledge of different consent types, biobanking, privacy, data use as well as their assessment and testing format preferences. Another strength was the systematic qualitative analysis of open responses that provided more profound insights into critical comments, concerns, and suggestions participants had.

Limitations include the fact that the random population-based study sample was biased towards older people, women, and participants with high levels of education compared to the general population aged 20 to 69 years. In addition to that, respondents participated in the SHeS-pilot. Thus, they tended to have supportive attitudes toward health research, which would not necessarily represent the perceptions and motivations of those who did not take part. Future research is needed to address issues identified in this study: (1) SHeS and other similar studies should consider potential impacts of findings from biomonitoring on cohort participants’ daily lives and be cautious about not reproducing inequalities through individual responsibility, as identified in the phenomenon of “precautionary consumption” [37], or non-intentional blaming-the-victim effects. It needs precaution regarding aggregated and individual results, as some findings can be adequately interpreted at the group or population level but are more difficult to interpret at the individual level. (2) A more nuanced understanding is needed of the boundaries between scientific findings at the population level and individual health consequences as well as data circulation across different spheres. The “return of results”, whether incidental findings or general analyses, may also play a role in people’s willingness (or not) to share their data and samples. However, the social and personal implications of genomic analyses, human biomonitoring results, and incidental findings are broad and often not anticipated by cohortees [46, 50]. (3) Future studies should also address those who declined to participate and focus on a better understanding of unwillingness and, ultimately, meaning of non-participation as those might differ from the meaning of and motivation for participation.

## Conclusions

Our study provides critical insights into issues central to building a cohort in personalized health research in Switzerland. We used a new scenario-based approach to explore willingness to participate, attitudes towards health research and biomonitoring, preferences regarding consent types, and data sharing. Participants showed high levels of willingness for participation, across various socio-demographic groups and diverse samples (random, purposive, self-selection). We also showed the usability t of open-ended comments in such a survey. Through those, participants shared perceived challenges, opportunities, and limitations in personalized health research. They expressed expectations from SHeS as well as concerns and reasons for non-participation in such a personalized health cohort. Participants also requested opt-out options, adjustments in devices and testings, and procedural clarity. Finally, they provided recommendations on how to improve content, form, and wording of the questionnaires.

Ultimately, these insights do not only provide valuable guidance for the implementation and enhancement of SHeS but also re-emphasize the relevance of aligning study methodologies with participant preferences to foster sustained engagement and maximize data quality.

### Electronic supplementary material

Below is the link to the electronic supplementary material.

Supplementary Material 1

Supplementary Material 2

Supplementary Material 3

## Data Availability

All relevant data are within the paper and its supplementary files.
