# Supplementary material for: Willingness to participate in a personalized health cohort – insights from the swiss health study pilot phase
Source: BMC Public Health. 2024 Aug 7;24:2140. doi: 10.1186/s12889-024-19650-z (PMC11305038; doi:10.1186/s12889-024-19650-z)
Supplement: Supplementary file 1 — Supplementary Material 1 [file 12889_2024_19650_MOESM1_ESM.docx]

**
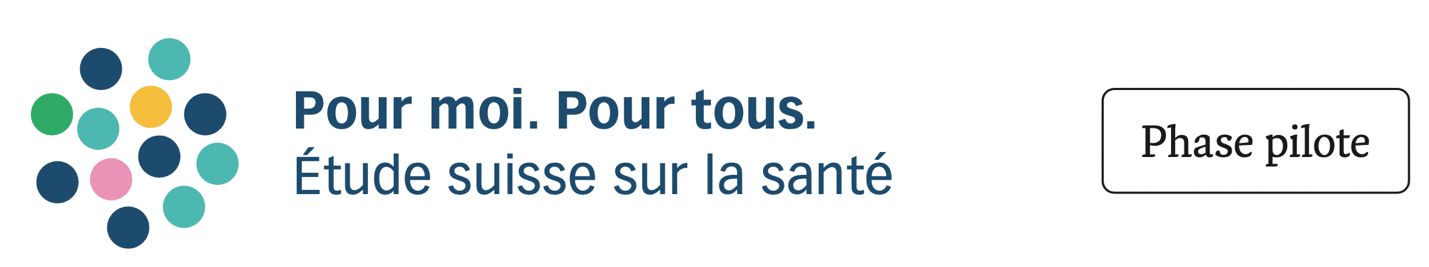
**

**Attitude towards**

**medical research**

**Attitude towards**

**medical research**

**About yourself**

| **1** | **You are:** |
| --- | --- |

☐ Female

☐ Male

| **2** | **What is your year of birth?** |
| --- | --- |

Year: _____________

| **3** | **What is your nationality?** |
| --- | --- |

☐ Swiss

☐ Dual nationality (namely): _________________ and ________________

☐ Foreign nationality (namely): ___________________________________

| **4** | **Which language(s) do you speak at home?** |
| --- | --- |

*Multiple answers possible.*

☐ French

☐ Swiss German

☐ German

☐ Italian

☐ Other (please specify): __________________________

| **5** | **How long have you lived in Switzerland?** |
| --- | --- |

☐ From birth

☐ Since _____________ (year(s))

| **6** | **What is your current housing situation?** |
| --- | --- |

☐ I live by myself

☐ I live with a partner/family (even if only temporarily, e.g., in case of shared custody)

☐ I live in a shared apartment/community accommodation

☐ Other living situation

(please specify): __________________________________________________

| **7** | **What is your highest level of education?** |
| --- | --- |

*Please check only one box.*

☐ Primary school

☐ Secondary school

☐ High school

☐ Apprenticeship / professional baccalaureate

☐ University degree: Bachelors

☐ University degree: Masters/License

☐ University degree: Doctorate/ PhD

☐ Other (please specify): _______________________________________________

☐ I do not wish to answer

| **8** | **Approximately, what is your household's total net monthly income?** |
| --- | --- |

*One answer only.*

☐ < CHF 3‘000

☐ between CHF 3‘000.- and CHF 4‘500.-

☐ between CHF 4‘500.- and CHF 6‘000.-

☐ between CHF 6‘000.- and CHF 9‘000.-

☐ between CHF 9‘000.- and CHF 11‘000.-

☐ > CHF 11‘000

☐ I do not wish to answer

| **9** | **Information concerning your profession:** |
| --- | --- |

1 What profession are you currently practicing? ______________________________________

2 What profession have you been trained for? _______________________________________

| **10** | **Do you belong to a religious denomination?** |
| --- | --- |

☐ Yes

Which one?

☐ Christian Catholic

☐ Christian Protestant

☐ Islam

☐ Judaism

☐ Other (please specify): __________________________

☐ No

☐ I do not wish to answer

| **11** | **How would you describe your general health status?** |
| --- | --- |

*One answer only.*

☐ Very good

☐ Good

☐ Average

☐ Poor

☐ Very poor

**Your opinion on health research**

Your opinion on health research and the questions it raises is very important to us. We are in the process of preparing a nationwide health study and would like to know the public's opinion on this subject.

To do this, we'd like you to put yourself in the shoes of Mrs. Martin, a fictional person. This will give you a more concrete idea of what things might be like if you were asked to take part in such a study.  **There are no right or wrong answers, it is YOUR opinion that counts**!

*Mrs. Martin receives a letter from health researchers. The researchers ask her if she'd be interested in taking part in a major health study that should lead to better prevention or treatment of certain diseases.*

| **12** | **In this situation, what is your first impression when you are told about this health study? Generally speaking…** |
| --- | --- |
|  |  |

☐ I would be very much in favour

☐ I would be somewhat in favour

☐ I would be somewhat against it

☐ I would be very much against it

☐ I am not concerned, or I don't care

| **13** | **Have you ever taken part in a health study?** |
| --- | --- |

☐ Yes

If so, please give us details about this study (e.g., study name, its objectives, your contribution)?

☐ No

☐ I don’t know

*The invitation letter inviting her to take part in the study lists the name and phone number of a contact person. Mrs. Martin calls and asks this person exactly how the study was conducted. She is told that the study involved filling in questionnaires and having blood and urine samples collected in a study centre.*

| **14** | **In this situation, would you agree to take part in a study involving the following actions:** |
| --- | --- |
|  |  |

|  |  | Yes | No |
| --- | --- | --- | --- |
| 1 | Completing a detailed questionnaire on certain risk factors and about your health | ☐ | ☐ |
| 2 | Going to a study centre for a health  examination (e.g., cantonal hospital or research centre) | ☐ | ☐ |
| 3 | Provide a blood, urine, or saliva sample | ☐ | ☐ |

*Mrs. Martin wonders whether this study is really useful and of good quality. Above all, she would like to know whether she is risking anything by taking part. To address this question, the contact person informs her that the study has been approved by her canton's ethics committee.*

| **15** | **Have you ever heard the term "ethics committee"?** |
| --- | --- |

☐ Yes, and I could explain the term

☐ Yes, but I don't know exactly what it means

☐ No

An ethics committee is a group of independent experts who assess the scientific, moral, social, and legal aspects of research projects. Any research project involving human beings must be approved by an ethics committee beforehand.

| **16** | **To what extent do the following aspects contribute to your confidence and trust in health research, or in a specific study?** |
| --- | --- |
|  |  |

|  |  | Very | Somewhat | Barely/not at all |
| --- | --- | --- | --- | --- |
| 1 | Being informed in a transparent and comprehensible manner about the progress of the research and its results | ☐ | ☐ | ☐ |
| 2 | Having the possibility of determining who may obtain my data | ☐ | ☐ | ☐ |
| 3 | Having the opportunity to meet the research team | ☐ | ☐ | ☐ |
| 4 | Approval of the study by an ethics committee | ☐ | ☐ | ☐ |
| 5 | To be able to express my opinion on the conduct of the study and to know that it will be taken into account, whenever possible. | ☐ | ☐ | ☐ |

*Mrs. Martin agrees to take part in the study and signs an informed consent form. By signing, she agrees to share with the researchers:*

- *Her* ***health data****: this data is collected using questions about her health status, medical records and living situation. This information should make it easier to prevent and treat certain diseases.*
- *A* ***sample*** *of her blood and other biological fluids (urine, saliva) for research into the biological mechanisms of certain diseases.*

*Mrs. Martin would like to know whether her name will be disclosed to the researchers and who will have access to her health data and biological samples.*

**There are three options for handling Mrs Martin's name and identity during a study:**

1. **Unencrypted data**: The researchers know the name and date of birth of the participants and, like the doctors, are subject to medical confidentiality.
2. **Encrypted data**: The researchers don't know whose data or blood they are analysing. However, in the interests of Mrs Martin (e.g., when the results are important for her health), the encryption of the data can be reversed. This can only be done with Mrs Martin's prior consent.
3. **Anonymised data**: The researchers do not know who the data or samples belong to and they have no way of determining which data and samples originate from Mrs. Martin. They can no longer provide Mrs Martin with information about her personal data, or only with exceptional effort.

| **17** | **Would you make your health data available to research, should they be in the following form?** | | | | |
| --- | --- | --- | --- | --- | --- |
|  |  |  |  |  |  |
|  | |  | Yes | Possibly | No |
| 1 | | Unencrypted data | ☐ | ☐ | ☐ |
| 2 | | Encrypted data | ☐ | ☐ | ☐ |
| 3 | | Anonymised data | ☐ | ☐ | ☐ |

| **18** | **Would you share your biological samples (blood, urine, saliva), should they be in the following form?** | | | | |
| --- | --- | --- | --- | --- | --- |
|  |  |  |  |  |  |
|  | |  | Yes | Possibly | No |
| 1 | | Unencrypted data | ☐ | ☐ | ☐ |
| 2 | | Encrypted data | ☐ | ☐ | ☐ |
| 3 | | Anonymised data | ☐ | ☐ | ☐ |

*On the phone, the contact person explains to Mrs. Martin that the health study she was being invited to includes a section on genetics (the study of genes). Mrs. Martin was informed that genetic tests are only relevant to her health in very rare cases and that only a small number of illnesses have a purely genetic explanation. Other factors, such as the environment and lifestyle, can also influence their development. The contact person explains that genetic analysis is useful in gaining a better understanding of the emergence of these diseases.*

| **19** | **In this situation, what is your first impression of genetic research?** |
| --- | --- |

☐ I'm very much in favour

☐ I'm somewhat in favour

☐ I'm somewhat against it

☐ I'm very much against it

☐ I'm not concerned by genetic research, or I don't care

| **20** | **If you were asked: in principle, would you be willing to participate in a study involving genetic issues?** |
| --- | --- |
|  |  |

☐ Yes

☐ No (Please state the reason here): __________________________________

☐ I don't know

*Mrs. Martin agreed to provide the researchers with a tube of blood. For the study in question, only part of the sample is analysed immediately, while the rest is kept in a 'biobank' for future research.*

| **21** | **Have you ever heard the term "biobank"?** |
| --- | --- |

☐ Yes, and I could explain the term

☐ Yes, but I don't know exactly what it means

☐ No

A biobank is a facility for storing and managing biological samples (e.g., blood, urine, saliva) and their associated data. Participants' samples are stored (anonymously) for several years and made available for scientific research. Analysing such a large number of samples provides a better understanding of the factors involved in the development of individual health. The greater the number of samples, the greater the potential for understanding the links between risk factors and disease.

| **22** | **Would you welcome the establishment of a national biobank in Switzerland for research purposes?** |
| --- | --- |
|  |  |

☐ Yes, definitely

☐ Rather yes

☐ Rather no

☐ No, not at all

☐ I don't know

*Mrs. Martin agrees to her blood being stored in a biobank along with the health data collected at the study centre. The contact person explains to Mrs. Martin that it is she who gives her agreement (consent) to the various ways in which the samples and associated data will be used. To do this, she signs a declaration of consent, which can take various forms. Mrs. Martin is also told that she can change her mind and withdraw her agreement to the use of her blood and associated data at any time.*

There are several forms of consent:

**General consent**: Mrs. Martin can give her consent just once, which allows the research teams to use the blood samples for any research project that has been validated by an ethics committee.

**Specific consent**: Mrs. Martin may be contacted again for each new research project, so that she can decide whether or not she agrees to her blood samples being used.

**Dynamic consent**: Mrs. Martin can decide at the time of the blood draw for which type of research project(s) her blood samples may be used. This option also allows her to change her preferences over time.

| **23** | **In the occurrence of a large national health study, what form of consent would you give?** |
| --- | --- |
|  |  |

Bearing in mind that you could withdraw or change your consent at any time and request that your samples be destroyed.

☐ General consent

☐ Specific consent

☐ Dynamic consent

☐ I would not donate biological material for research

☐ I don’t know

*Over the phone, the contact person also informs Mrs. Martin that an "electronic patient record" had recently been introduced in Switzerland. This allows patients to collect and centralise their medical data and manage it themselves.*

| **24** | **Are you familiar with the term "electronic patient record"?** |
| --- | --- |

☐ Yes, and I could explain the term

☐ Yes, but I don't know exactly what it means

☐ No

The electronic patient record is the collection of personal information, data and documents relating to a person's health on a secure Internet platform. This information can be consulted at any time by medical staff, with the consent of the individual concerned. The electronic patient record could help researchers in a health study to understand the link between a current or future illness and certain substances detected in samples.

| **25** | **Can you envisage opening an electronic patient file?** |
| --- | --- |

☐ I already have an electronic patient record

☐ Yes

☐ No

☐ I don’t know

| **26** | **At present, there are no intent to use electronic patient records for research purposes. However, if you had an electronic patient record, would you be willing to give researchers access to information in this record?** |
| --- | --- |
|  |  |

(As a patient, you decide which information could be accessed)

☐ Yes

☐ No

☐ I don’t know

**Willingness to participate in a long-term health study**

There are different forms of health research, and we'd like to hear your views on long-term health studies (also known as "longitudinal cohort studies"), in which participants are monitored over a long period of time (questionnaires, medical visits) to observe changes in their health status. Why does one person fall ill while another remains in good health? What promotes the onset of disease? What role do the environment, social context, diet, or genetic predisposition play?

These are the questions that long-term studies aim to answer, in order to improve disease prevention, diagnosis and treatment. Here again, your opinion is invaluable, as it will help us to set up a new long-term study in Switzerland, the Swiss Health Study.

| **27** | **In principle, would you be willing to participate in a longitudinal study?** |
| --- | --- |

☐ Yes, definitely

☐ Rather yes

☐ Rather no

☐ No, not at all

☐ I don’t know

Why not? ___________________________________________________

| **28** | **How would you like to be contacted as a first instance about participating in a longitudinal study?** |
| --- | --- |

*Please tick all that apply.*

☐ By postal letter

☐ By phone

☐ Via Internet (e-mail, social media, etc.)

☐ By a home visit from the research team

☐ At my GP/family doctor's office

☐ At the pharmacy

☐ At the hospital

☐ Other option(s): __________________________________________________

| **29** | **Should you agreed to take part in a longitudinal study, how would you like to answer the study questions?**  *Please tick all that apply.* |
| --- | --- |
|  |  |

☐ In writing (via questionnaires)

☐ By telephone (as an interview)

☐ Online via the Internet

☐ By a personal interview at your home

☐ By a personal interview at a study centre

☐ On your Smartphone (mobile phone) via an app

☐ Other option(s): __________________________________________________

| **30** | **What is/would be your motivation to participate in a longitudinal study and donate time, information and, if necessary, biological material?**  *Please tick all that apply.* |
| --- | --- |
|  |  |

☐ I would like to contribute to medical progress

☐ I would like to contribute to improving the health of other people

☐ I am interested in research and health

☐ I would like to benefit from a free medical check-up

☐ I am interested in knowing the results of the study

☐ I am proud to be taking part in an important study in Switzerland

☐ I am motivated by financial remuneration

☐ I am motivated by small gifts (e.g., REKA cheque, travel vouchers)

☐ Other possibility(s): __________________________________________________

☐ No reason would motivate me

| **31** | **For which reasons would you refuse to participate in a longitudinal study?** |
| --- | --- |

*Please tick all that apply.*

☐ I'm not interested at all

☐ I don't have the time

☐ I'll only have time to take part in the evenings or during weekends

☐ I'll never benefit personally from the results

☐ I'm not in favour of health research

☐ I don't think such study could help improve the health of the population

☐ I don't want to travel to the study centre

☐ I don't want to share my health data

☐ I don't want to donate blood (or other biological samples)

☐ I'm afraid my data won't be properly protected

☐ I'm afraid my data might be misused (e.g., by health insurance companies, employers, etc.)

☐ I fear that my contribution will serve the private interests of the pharmaceutical industry

☐ Other possibility(s):___________________________________________________

☐ I would not refuse

| **32** | **Which biological samples would you donate to research as part of a longitudinal study (all are equally important and valuable for research)?**  *Please tick all that apply.* |
| --- | --- |
|  |  |

☐ Saliva

☐ Hair

☐ Urine

☐ Blood

☐ Stool

☐ Genetic material (DNA) (e.g., from a blood or saliva sample)

☐ None of the above

| **33** | **What types of examination would you be willing to take as part of a longitudinal study?**  *Please tick all that apply.* |
| --- | --- |
|  |  |

☐ Simple anthropometric measurements (e.g., height, weight, waist circumference, arm circumference)

☐ Measurement of body functions (e.g., blood pressure, bone density measurement, ECG)

☐ Wearing of small measuring devices for a defined period of time (e.g., 24-hour blood pressure, measurement of physical activity over 1 week, etc.)

☐ Physical capacity tests (e.g., handgrip test, lung capacity, mobility tests)

☐ Medical imaging (e.g., ultrasound, MRI)

☐ Questionnaires on specific topics (e.g., diet, use of chemicals)

☐ Blood count

☐ Sensory analysis (sight, smell, hearing, taste)

☐ Cognitive tests (e.g., memory)

☐ Collection of urine over 24 hours at home with organized transport to the study centre

☐ Collection of a drop of blood from the finger on blotting paper (equipment provided) and sent by post via pre-stamped postal envelope

☐ Collection of stool at home (simple and hygienic collection system) with organised transport to the study centre

☐ Collection of samples at the research centre in your region

☐ Diagnostic tests (e.g., allergy tests, diabetes)

| **34** | **All these examinations can provide important information about your health, with some illnesses having treatment or prevention possibilities, others not directly. What type of results would you be interested in knowing, if possible?** |
| --- | --- |
|  |  |

|  |  | Yes | No |
| --- | --- | --- | --- |
| 1 | The results of a simple medical check-up carried out during your visit to the study centre (high blood pressure, suspected diabetes, and simple laboratory tests, e.g., high cholesterol) | ☐ | ☐ |
| 2 | Results of your environmental exposure (e.g., pesticides, plastic compounds, heavy metals) | ☐ | ☐ |
| 3 | Genetic findings, should they indicate the risk of preventable diseases | ☐ | ☐ |
| 4 | Genetic findings, should they indicate the risk of treatable diseases | ☐ | ☐ |
| 5 | Genetic findings, should their be a chance they could have an impact on my (future) children | ☐ | ☐ |

| **35** | **How would you like these results to be communicated to you?** |
| --- | --- |

*Please tick all that apply.*

☐ I don't want to know the study results

☐ I want to know the study results

Please specify :

|  |  | General results from examinations and tests (except genetic tests) | Genetic Findings |
| --- | --- | --- | --- |
| 1 | Per letter/mail | ☐ | ☐ |
| 2 | Per e-mail | ☐ | ☐ |
| 3 | Per phone | ☐ | ☐ |
| 4 | During a medical consultation with my GP or family doctor | ☐ | ☐ |
| 5 | During a visit by the research team | ☐ | ☐ |

The aim of the planned longitudinal study is to understand the influence of the environment on health, e.g., pollution by pesticides, plastics or noise, as well as our lifestyle habits (e.g., diet, physical activity).

| **36** | **How important do you think it is to study the possible effects of the environment on health?** |
| --- | --- |
|  |  |

☐ Very important

☐ Quite important

☐ Not so important

☐ Not important at all

☐ I don't feel concerned by this issue / I don't care

For some research studies, collaborations are desirable, as they can improve the results obtained through the contribution of other researchers, the pooling of several datasets, or the provision of additional funding to carry out additional analyses. Participant health data and/or biological samples from a biobank can then potentially be shared with researchers from institutions other than the one that carried out the research in the first place, in Switzerland or abroad. Data is only exchanged with the consent of the participant and in compliance with Swiss legal provisions, particularly in terms of data protection.

| **37** | **Which researchers or institutions would you authorise to use your data and biological samples for research?**  *Multiple answers possible.* |
| --- | --- |
|  |  |

☐ Researchers from Swiss universities or clinics

☐ Researchers from foreign universities and clinics

☐ Researchers from federal offices (researchers subsidised by the government)

☐ Researchers from non-profit organisations (e.g. Ligue contre le cancer)

☐ Researchers from the pharmaceutical industry

☐ Researchers from the agri-food industry

☐ Researchers from the fitness industry

☐ I would not like to share my biological samples with researchers

| **33** | **In the context a longitudinal study, would you allow the researchers in charge of the study to request access to your health information from the following sources?**  *Please note that this will not be done for the pilot phase of the Swiss Health Study.* |
| --- | --- |
|  |  |

|  |  | Yes | Possibly | No |
| --- | --- | --- | --- | --- |
| 1 | General practitioner/Family doctor | ☐ | ☐ | ☐ |
| 2 | Specialists (gynecologist, dermatologist, ophthalmologist....) | ☐ | ☐ | ☐ |
| 3 | Other medical partners (pharmacy, Spitex/Ambulatory care, nursing home) | ☐ | ☐ | ☐ |
| 4 | Hospital | ☐ | ☐ | ☐ |
| 5 | Databases of institutions and medical organizations | ☐ | ☐ | ☐ |
| 6 | Health and diagnostic registers (e.g. tumour registers) | ☐ | ☐ | ☐ |

The origin of an illness is often complex, and several factors may come into play. In order to study the origins of an illness, all the factors that influence health must be identified. For this reason, the questionnaires for a long-term study can be relatively long, but they can be completed in several stages.

| **39** | **What is a reasonable time commitment for you to complete a questionnaire (paper or online) about your health and life circumstances?** |
| --- | --- |
|  |  |

☐ 4 hours or more

☐ 2 - 4 hours

☐ 1 - 2 hours

☐ Less than 1 hour

☐ I don't know / it would depend on the questions

A visit to the study centre is used to carry out a detailed assessment of a person's state of health. Numerous parameters and symptoms of potential illnesses must be recorded. The visit to the study centre may therefore take some time, depending on the examinations performed.

| **40** | **How much time would you agree to dedicate to examinations at a study centre (incl. travel, examination, sample collection)?** |
| --- | --- |
|  |  |

☐ A whole day

☐ Half a day

☐ 1-2 hours

☐ I don't know

| **41** | **In some studies, study participants are involved in the decision making regarding the study. What is your opinion on this?** |
| --- | --- |
|  |  |

☐ I don't want to be involved

☐ I would like to be informed regularly, for example by means of a newsletter

☐ I would like to express my opinion about decisions concerning the study

☐ I would like to take an active part in shaping the research framework in a discussion group

☐ I don't know

☐ I would like to contribute in some other way

(please specify): ____________________________________________________

| **42** | **Would you be interested in being involved in decisions about this study, as part of a focus group organised by the research team?** |
| --- | --- |
|  |  |

☐ Yes

☐ No

| **42.1** | **If you would like to be part of such a group, please let us know how you would like to be contacted:** |
| --- | --- |
|  |  |

_______________________________________________________________

_______________________________________________________________
