# Supplementary figures and images for: Willingness to participate in a personalized health cohort – insights from the swiss health study pilot phase
Source: BMC Public Health. 2024 Aug 7;24:2140. doi: 10.1186/s12889-024-19650-z (PMC11305038; doi:10.1186/s12889-024-19650-z)

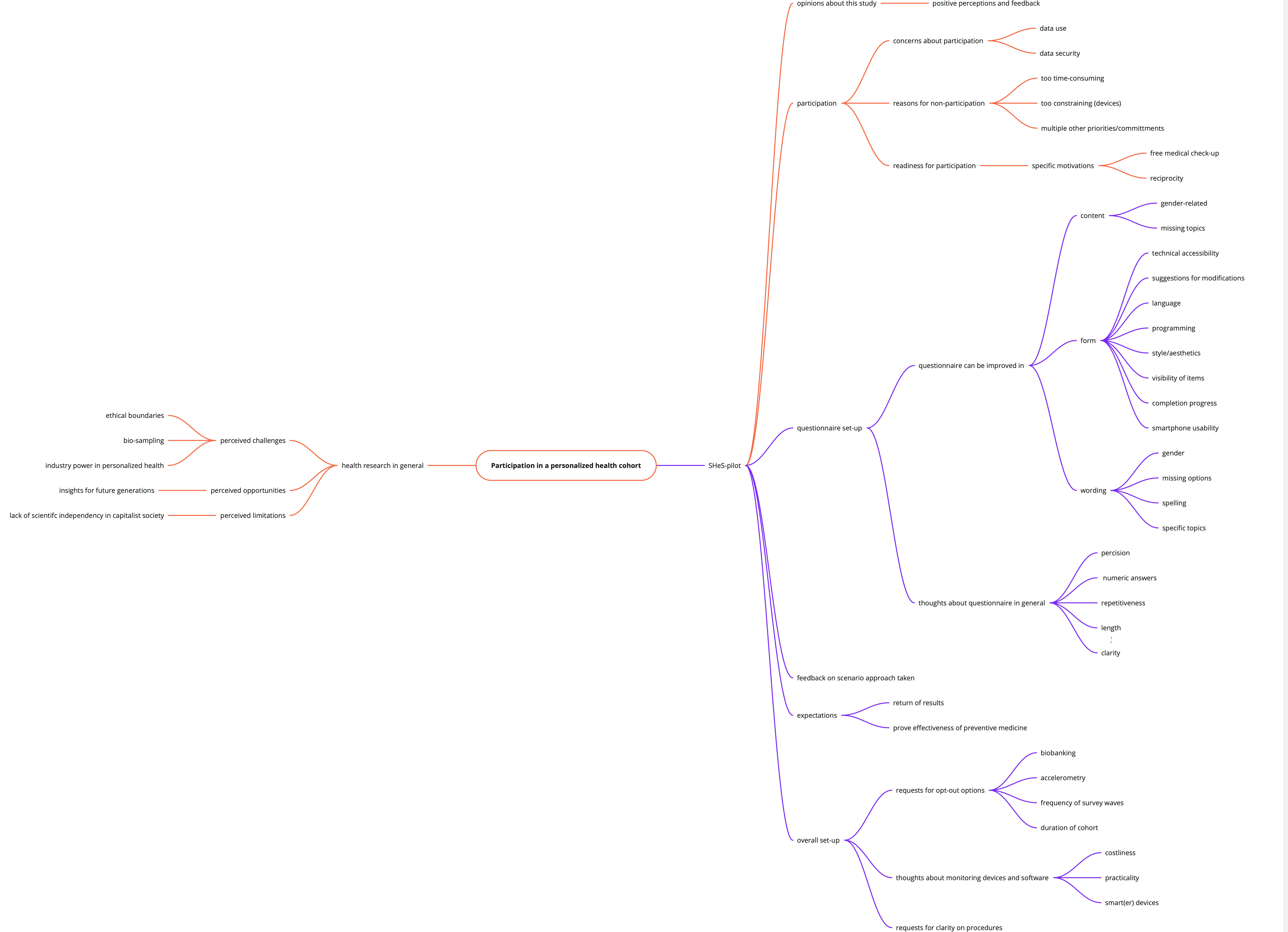

Supplement: Supplementary file 2 — Supplementary Material 2 [file 12889_2024_19650_MOESM2_ESM.jpg]
